# Hydrogels for Hydrophobic Drug Delivery. Classification, Synthesis and Applications

**DOI:** 10.3390/jfb9010013

**Published:** 2018-01-24

**Authors:** Eneko Larrañeta, Sarah Stewart, Michael Ervine, Rehan Al-Kasasbeh, Ryan F. Donnelly

**Affiliations:** Queens University Belfast, School of Pharmacy, 97 Lisburn Road, Belfast BT9 7BL, UK; sstewart35@qub.ac.uk (S.S.); mervine01@qub.ac.uk (M.E.); ralkasasbeh01@qub.ac.uk (R.A.-K.); r.donnelly@qub.ac.uk (R.F.D.)

**Keywords:** hydrogels, drug delivery, hydrophobic drugs, micelles, nanoparticles, physical hydrogels

## Abstract

Hydrogels have been shown to be very useful in the field of drug delivery due to their high biocompatibility and ability to sustain delivery. Therefore, the tuning of their properties should be the focus of study to optimise their potential. Hydrogels have been generally limited to the delivery of hydrophilic drugs. However, as many of the new drugs coming to market are hydrophobic in nature, new approaches for integrating hydrophobic drugs into hydrogels should be developed. This article discusses the possible new ways to incorporate hydrophobic drugs within hydrogel structures that have been developed through research. This review describes hydrogel-based systems for hydrophobic compound delivery included in the literature. The section covers all the main types of hydrogels, including physical hydrogels and chemical hydrogels. Additionally, reported applications of these hydrogels are described in the subsequent sections.

## 1. Introduction

Hydrogel technology has increased in popularity ever since the original work on synthetic crosslinked 2-Hydroxyethyl methacrylate (HEMA) hydrogels was carried out by Lim and Wichterle in the late 1950s [1]. Hydrogels are defined as three-dimensional crosslinked hydrophilic polymeric networks that are capable of imbibing up to thousands of times their dry weight in water or biological fluids [2,3]. Due to the high water content and the physiochemical similarity to the native extracellular matrix, hydrogels are highly biocompatible [4]. This is verified by their positive use in the peritoneum [5] and other sites in vivo. To undergo successful sol-gel transformation, hydrogels may be fine-tuned to be receptive to varying environments present in the body such as pH, temperature, and enzymatic activities at the diseased sites [6]. Body temperature (37 °C) [7] and physiological pH (7.4) [8] are commonly the conditions in which a hydrogel transforms from the sol-state to the gel-state.

Hydrogels are often used for the delivery of hydrophilic drugs. However, as the polymer matrix is hydrophilic, hydrophobic drugs are generally incompatible with hydrogels [9]. This is due to hydrophobic drugs having limited loading quantity and homogeneity in hydrogel matrices [10]. Therefore, hydrogels should be adapted to be able to deliver hydrophobic compounds. This is major challenge as hydrophobic drugs are playing an important role in current pharmaceutical treatment. It is estimated that about 40% of the marketed drugs and 60% of the compounds at research and development state present poor water solubility [11].

Two of the main approaches to improve the compatibility of hydrogels with hydrophobic compounds are the introduction of molecules capable of forming inclusion complexes (i.e., cyclodextrins) and/or the incorporation of hydrophobic moieties in the hydrogel structure [10,12]. However, this is not the main type of hydrogel used for poorly water soluble drug delivery. More sophisticated systems have been described including hydrogels containing micelles/nanoparticles within their structure [10,12].

In this review, we will focus on the different strategies described in the literature to effectively incorporate hydrophobic drugs within hydrogels. The first part of the review describes the different strategies used to prepare hydrogels compatible with hydrophobic compounds. Subsequently, the second part of the review is focused on the main applications of these materials as drug delivery systems.

## 2. Types of Hydrogels

### 2.1. Physical Hydrogels

**Figure 1:**
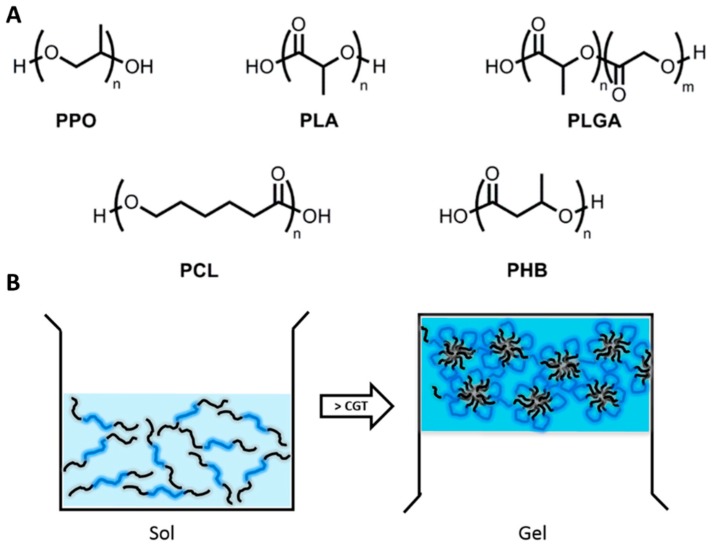
Hydrophobic polymer blocks in thermosensitive gel-forming triblock copolymers. PPO: Poly(propylene oxide), PLA: Poly(d,l-lactide), PLGA: Poly(d,l-lactide-co-glycolide), PCL: Poly(ε-caprolactone), PHB: Poly[(R-3-hydroxybutyrate] (**A**). Schematic illustration of a sol-to-gel transition of PLGA-PEG-PLGA triblock copolymers. CGT: Critical gelation temperature (**B**). Reproduced with permission from [10].

Thermosensitive hydrogels respond to changes in temperature and usually undergo a sol-gel phase transition when the temperature changes from room to physiological temperature. This makes them particularly useful, as temperature is generally an easy stimulus to control [13]. Thermosensitive hydrogels are usually triblock polymers made up from poly(ethylene glycol) (PEG) linked to hydrophobic polymer blocks. The triblock is composed of A blocks and B blocks organised as ABA or BAB [13]. PEG as the A block, is well-established for its use in hydrogel formulation as it possesses high water solubility, biocompatibility and low immunogenicity [14]. While the B blocks increase the hydrophobicity and drug loading capacity of hydrophobic drugs by micellization. Some of the common block copolymers used for the preparation of physical hydrogels are shown in Figure 1A.

The most widely used reverse thermal gelation polymers are the triblock copolymers made up of PEG as the A Block and poly(propylene oxide) (PPO) as the B block arranged as ABA, [15] known as poloxamers or pluronics. Poloxamers show exceptional thermogelling behaviour. Poloxamer 407, a well-known example, has been shown to be a viscous liquid at room temperature or below but forms a hydrogel at body temperature (37 °C) [16]. A previous study [17] has shown poloxamer 407 to act as a drug depot for the relatively hydrophilic lidocaine, promoting extended duration of release. However, loading of hydrophobic drugs is limited due to the gel consisting of two hydrophilic PEG blocks sandwiching only one hydrophobic PPO block. To improve this shortfall, a method employing alpha-cyclodextrin to form crystalline inclusion complexes called polypseudorotaxanes was used [18]. This procedure enhanced the loading of hydrophobic drugs and moreover, prolonged the release period. It important to note that poloxamer hydrogels have a high critical micelle concentration (CMC), which has restricted their use in a wide range of medical fields. The high CMC is caused by the weak hydrophobic interaction between PPO blocks meaning a higher concentration of triblock copolymers is required to form micelles. This is problematic as hydrogels possessing a high CMC may cause a rapid physical dissociation, causing a burst effect after administration due to the dilution of hydrogels with a large volume of physiological fluid. This issue may be surmounted by substituting PPO for a more hydrophobic B block.

An alternative thermogelling hydrogel is the triblock copolymers consisting of PEG and poly(lactic acid) (PLA) disposed in the following way: PLA-PEG-PLA [19]. These hydrogels have been significantly studied and are used widely for drug delivery applications due to their biodegradability and biocompatibility properties and their valuable ability to self-assemble in aqueous media to form polymeric micelles with a core–shell structure [20]. One study used ultraviolet irradiation to photo-crosslink PLA-PEG-PLA hydrogels with acrylated end groups [19]. They proved that by altering the concentration of crosslinker, ethylene glycol dimethylacrylate (EGDMA), or changing the UV irradiation time, the size of the nanogel could be easily regulated between the range of 150–250 nm. Using camptothecin as the hydrophobic model drug, they indicated that the nanogel has a high encapsulation efficiency (80%) and has a controllable release rate over at least 20 days. A further positive study [20] has been carried out on the PLA-PEG-PLA triblock copolymers. Though, on this occasion the gels were not photo-crosslinked but instead were synthesised by the nanoprecipitation method, with the nanogel being formed by thermal crosslinking [20]. This study illustrated how changing the polymer concentration, the size of nanogels could be controlled between the range of 128–200 nm. This time using naltrexone as the hydrophobic model drug, the encapsulation efficiency was shown to be high at 60% and could sustain the release of naltrexone for up to 35 days, depending on the crosslinker (EGDMA) concentration used. As a result, both these hydrogels/nanogels show promise for the sustained release of hydrophobic drugs.

Using PLA as the hydrophobic B block has proven to show some issues [21]. To overcome these problems and enhance the properties of PLA, an approach has been to lower the hydrophobicity by copolymerising lactide with the more hydrophilic glycolide to create Poly(lactic co glycolic acid) (PLGA) [21]. One well-recognised thermosensitive hydrogel is called ReGel^®^. It consists of a triblock copolymer arranged as PLGA-PEG-PLGA and is a free flowing water soluble solution at low temperatures (2–15 °C) but transitions to a gel at body temperature (37 °C) [22] (Figure 1B). A study performed on the alternate arrangement PEG-PLGA-PEG, found these hydrogels had similar properties to ReGel^®^, i.e., sol-gel transition temperature of 37 °C [13].

In an investigation carried out on hydrogels consisting of the triblock copolymer arranged as PEG-PLGA-PEG [23], it was discovered that the release profiles of drugs varied depending on their hydrophobicity. For instance, when the gels were loaded with relatively hydrophilic (ketoprofen: Log *p* = 0.97) and hydrophobic (spironolactone: Log *p* = 2.78) drugs, the hydrophilic drug was released over two weeks, while the hydrophobic drug was released over two months.

As a result of thermosensitive hydrogels usually having a gelation transition temperature of 37 °C, this has caused problems during administration using a syringe/needle. This is because the patient’s body temperature causes the rapid gelation of the hydrogel and thus blocks the needle [8]. To overcome this barrier, researchers have added pH-sensitive moieties to existing thermosensitive copolymers, so that for gelation to occur a second condition must be met i.e., pH.

One approach to generate gels that are thermo- and pH-sensitive is though the addition of sulfamethazine oligomers (OSM) endcaps to already thermosensitive copolymers. An example of this is the addition of OSM endcaps to the thermosensitive parent triblock poly(ε-caprolactone-co-lactide)-b-poly(ethylene glycol)-b-poly(ε-caprolactone-co-lactide) (PCLA-PEG-PCLA) to form OSM-PCLA-PEG-PCLA-OSM [8]. When the formulation is at room temperature and at pH 8.0 it is in the sol-state, to transition to the gel-state, two conditions must be met, the temperature must rise to 37 °C and the pH must drop to pH 7.4. This means the needle will not become blocked during administration as only increasing the temperature will not cause the change to the gel form on its own, the pH must also drop to 7.4 inside the needle, which does not occur. Thus, this formulation may be employed as injectable carriers for hydrophobic drugs.

The triblock copolymers consisting of PEG as the A Block and poly(acrylic acid) PAA as the B block arranged as BAB has been shown to have a thermo- and pH-sensitive nature [13]. At relatively low pHs, e.g., pH 3.0, the PAA block is hydrophilic but, at higher pHs e.g., pH 7.4, the PAA block becomes hydrophobic. Furthermore, as the temperature increases, the pKa of the PAA-PEG-PAA polymer decreases, indicating that an increase in temperature, increases the hydrophobicity [13]. The PAA-PEG-PAA polymer was found to undergo a sol-to-gel-to-condensed gel transition at pH 7.4 and at 37 °C, with the condensed gel having a high viscosity of 43.6 kPa·s [13].

The addition of poly(β-amino ester) (PAE) endcaps to a PCL-PEG-PCL triblock imparts a pH-sensitive nature, in addition to the thermosensitive properties already held by the parent triblock [24]. The parent block alone would only transition in response to temperature (gel region of 34–54 °C) [24] but the addition of PAE endcaps to form a pentablock (PAE-PCL-PEG-PCL-PAE), leads to the hydrogel being sensitive to both pH and temperature changes [8]. This formulation was found to undergo sol-gel phase transition above pH 6.0 in response to increasing both temperature and pH [24].

### 2.2. Covalent Hydrogels

An alternative way to introduce hydrophobic domains within a hydrogel network is by cross-linking hydrophobic chains/monomers with hydrophilic ones. A similar effect can be obtained by using hydrophobic crosslinkers. These hydrogels are also called amphiphilic hydrogels. Several strategies can be taken to prepare this type of hydrogel.

**Figure 2:**
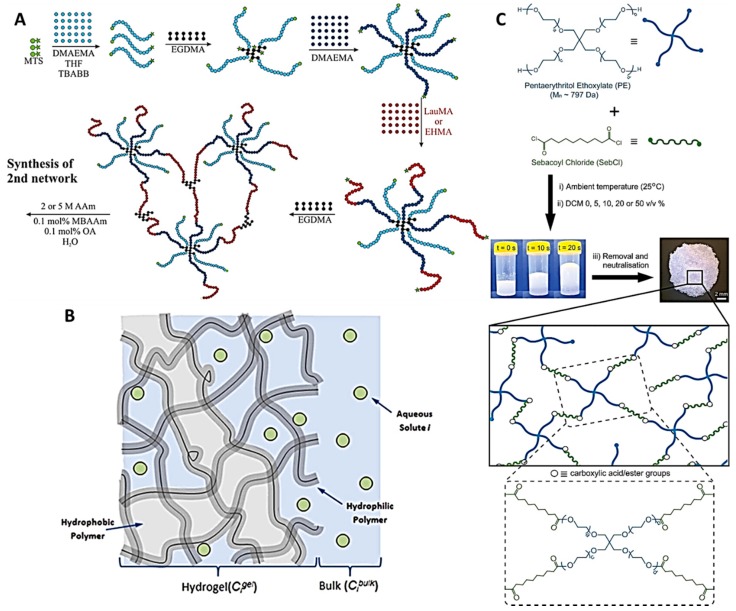
Schematic Representation of the Synthetic Procedure Followed by Rikkou-Kalourkoti et al. for the Preparation of the amphiphilic polymethacrylate conetwork First Network (**A**). Schematic illustration of the hydrogels developed by Liu et al. (**B**) Hydrogel synthesis developed using pentaerythritol ethoxylate and sebacoyl chloride (**C**). Reproduced with permission from [26,27,28]. Figure (**A**): Copyright (2016) American Chemical Society.

The literature contains several examples of the use of free-radical polymerization techniques to synthesize different types of polymers [25]. One of the typical examples is the formation of a co-network between poly(methacrylate) (PMA) (amphiphilic) with a second poly(acrylamide) (PAM) network [26] (Figure 2A). To prepare these hydrogels the first step was to obtain a hydrogel network made of methacrylate (MA) chains crosslinked using ethylene glycol dimethacrylate (EGDMA). Subsequently, the second network was formed inside the MA/EGDMA one using a photo-polymerization to crosslink acrylamide using N,N’-methylenebis(acrylamide) [26].

Silicon-based hydrogels obtained using free-radical polymerization are an alternative option to obtain hydrogels containing hydrophobic domains. This type of hydrogel is widely used for contact lens manufacturing [27] (Figure 2B). Amphiphilic silicone hydrogels can be obtained via thermally initiated free-radical polymerization of a silicone-grafted monomer, a MA-based hydrophilic monomer, a crosslinking agent, and an amphiphilic triblock-macromer [27]. The latter is required to prevent macrophase separation during the synthetic procedure due to the different nature of the monomers.

PEG-based polymers have been widely used to prepare covalently crosslinked hydrogels with hydrophobic domains. The modification of PEG chains to include hydrophobic chains to prepare this type of systems has been extensively studied. For this purpose, PEG was combined with the hydrophobic polymer polydimetylsiloxane (PDMS) using tetrahydrofuran as solvent and UV radiation [29,30,31]. The hydrogel properties were (swelling and mechanical properties) dependent on the PEG and PDMS content of the resulting product. A different synthetic procedure to obtain PEG-based amphiphilic hydrogels was proposed by Hamid et al. [32]. In this work poly(ethylene glycol) diglycidyl ether (PEGDGE) was combined with diamino crosslinkers such as cystamine and α, ω-diamino-PCL in dimethyl sulfoxide. On the other hand, Ozcelik et al. proposed a rapid process to prepare hydrogels by using hydroxyl end-functionalized 4-arm PEG to crosslink sebacoyl chloride hydrophobic chains [28] (Figure 2C). This hydrogel was obtained using an esterification reaction and consequently can be biodegraded. In a different work, Ozcelik et al. presented a transparent alternative of the previous hydrogel by inctroducing α, ω-dihydroxy-PCL in the system [33].

The use of condensation between anhydrides and alcohols has been used to synthesize hydrogels in a simple way. For example, pyromellitic anhydride was used a hydrophobic cross-linker due to the presence of anhydride groups in its structure that can react easily with OH groups in other polymer chains [34]. This strategy presents advantages over the previous ones, as it does not require an initiator, coupling agents or other additives that can present toxicity problems limiting the biomedical applications of the hydrogels. A similar type of approach was developed by Larrañeta et al. by using poly(methyl vinyl ether-alt-maleic acid) crosslinked with a surfactant (Tween^®^ 85) [35]. In this case the crosslinking process was carried out in solid state without using any type of solvent.

### 2.3. Nanoparticle-Containing Hydrogels

The inclusion of nanoparticles within the hydrogel structure is a good approach to include hydrophobic depots in the material. The nanoparticles can be covalently linked to the hydrogel structure or just simply absorbed inside the material. For the first approach, nanoparticles can be prepared containing polymerizable groups in their surface that can be copolymerized with hydrophilic momomers. Additionally, if the particles are multifunctionalised they can act as crosslinkers for the hydrogels. Using this approach, the diffusion of the particles during the swelling process can be prevented.

**Figure 3:**
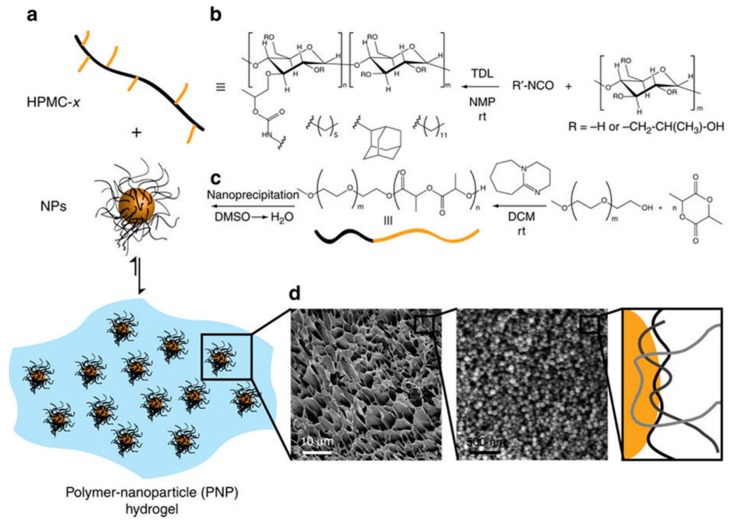
Schematic representation of the preparation of polymer–nanoparticle (PNP) hydrogels utilizing non-covalent interactions between core-shell nanoparticles (NPs) (**a**) and hydrophobically modified hydroxypropylmethylcellulose (**b**). The NPs can be composed of either poly(styrene) (PS; non-degradable) or poly(ethylene glycol)-block-poly(lactic acid) (PEG-b-PLA; biodegradable) (**c**). Cryogenic scanning electron microscopy images of PNP gels composed of polystyrene nanoparticles (*d* ~ 50 nm) demonstrate a homogeneous distribution of NPs within the gel structure, indicating that the network is held together by multivalent, dynamic polymer–nanoparticle interactions (as illustrated; polymer chains, grey scale; nanoparticle, orange) (**d**). Reproduced with permission from [39].

The more common approach is to incorporate the nanoparticles before the crosslinking process (physical or chemical). For this purpose, the particles should be prepared previously and added to the reaction mixture. In this way the particles can be easily incorporated to the hydrogel structure. However, they are not covalently attached to the structure and, consequently, they can diffuse out from the hydrogel during the swelling process. This approach was used to prepare contact lenses containing PCL-based nanoparticles for ocular drug delivery [36] (Figure 3). In this case a 2-hydroxy ethyl methacrylate (HEMA) based hydrogel was synthesized including the nanoparticles inside the hydrogel structure. Using methacrylates, Schoener et al. developed pH-responsive hydrogel containing poly(methyl methacrylate) (PMMA) nanoparticles for hydrophobic drug delivery [37]. For this purpose the hydrogel made of methacrylic acid grafted with poly(ethylene glycol) tethers was crosslinked including PMMA nanoparticles in the reaction mixture. In a similar way, Bini et al. developed a nanocomposite gel composed of gelatin and poly(3-hydroxybutyrate) (PHB) polymeric nanoparticles for curcumin delivery [38]. Gelatin was crosslinked by using an enzymatic procedure in the presence of nanoparticles.

Inorganic nanoparticles have been incorporated into hydrogels. Yang et al. described the incorporation of acrylic acid (AA) and octyl-phenol-oligo(oxyethylene)-acrylate to the surface of silicon nanoparticles [40]. These functionalized nanoparticles were used to prepare a network of poly(acrylic acid) (PAA) hydrophilic backbone chains linked to randomly distributed nanoparticles. The system included physical-crosslinking due to the presence of hydrophobic branches (octyl-phenol). Additionally, magnetic nanoparticles composed of Fe_3_O_4_ were used by Zhou et al. to prepare hydrogels [41]. The particles were incorporated to a PAA-based structure through coordination bonding with the carboxyl groups of PAA blocks.

Physical adsorption of nanoparticles to polymeric chains provides an alternative way to obtain hydrogels. The interaction of the particles with the polymeric backbone should fulfill a series of conditions to achieve hydrogels via nanoparticle-polymer physical interaction [39]. The particles should exhibit strong affinity for the polymer chains. Additionally, every polymer chain and nanoparticle should establish at least two interactions to achieve the percolation of the network. Finally, the particle diameter should be comparable to, or less than, the persistence length of the polymer strands. Following these rules, Appel et al. described the preparation of self-assembled hydrogels via the interactions between the chains of hydroxypropylmethyl-cellulose derivatives and PEG-poly(lactic acid) (PLA) nanoparticles [39]. In a similar way, poly(acrylate)-based hydrogels with incorporation of cross-linked styrene-butadiene-styrene particles were prepared by Cevik et al. [42].

### 2.4. Hydrogels Containing Cyclodextrins

Cyclodextrins (CDs) have been extensively used for complexation of hydrophobic molecules [11,43]. CDs are cyclic oligosaccharides formed by 6, 7 and 8 dextrose units (α, β, γ-CD, respectively) bound by 1–4 carbon bonds [11,43]. They have the structure of a truncated cone containing a hydrophobic cavity and a hydrophilic outer surface. The presence of the hydrophobic cavity enables the formation of inclusion complexes with hydrophobic molecules, thus enhancing the solubility of the molecule. Consequently, CDs have been extensively used in drug delivery [43].

**Figure 4:**
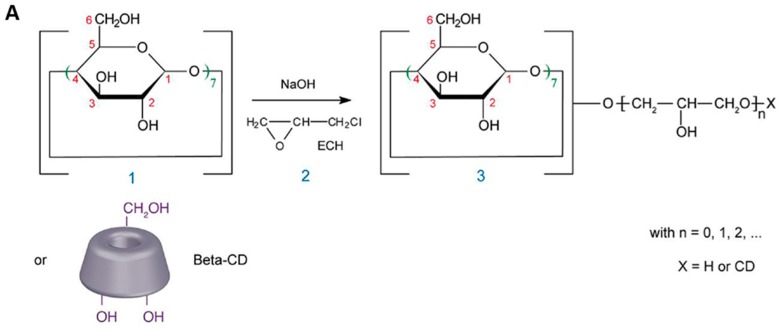

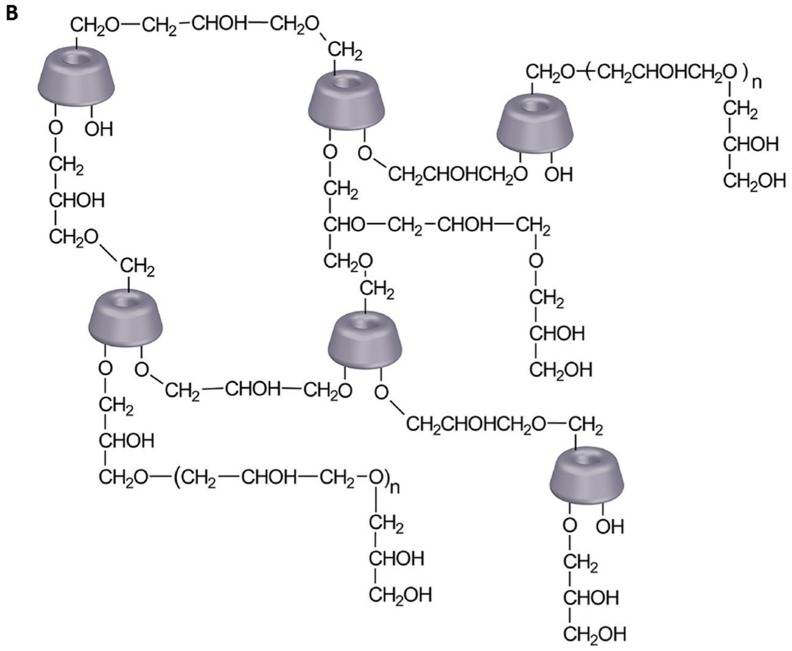
Chemical reaction between β-cyclodextrin (β-CD) and epichlorohydrin in basic medium (**A**). Structure of a water insoluble epichlorohydrin-cross-linked CD material (**B**). Reproduced with permission from [46].

CDs have been extensively used in the preparation of hydrogels to obtain materials with the ability to form inclusion complexes with hydrophobic drugs [43]. Many scientists have combined CDs with chemical crosslinkers such as epichlorohydrine (EP) (Figure 4), triazine, or diisocyanates [43,44,45]. These crosslinkers can react with the multiple OH groups present in CD molecules, yielding hydrogels.

CD-EP hydrogels have been loaded with different hydrophobic drugs [43]. Jug et al. used CD-EP hydrogels to load and release bupivacaine for sustained delivery of this anaesthetic in the buccal cavity [47]. In a similar way CD-EP were prepared by using cationic CDs for loading of antibiotic complexes [48]. On the other hand, diisocianates have been used to crosslink CD alone or in combination with PEG chains [43]. The crosslinking of CDs with triazine has been used to synthesize hydrogels for the delivery of drugs used for HIV treatment or hydrophobic anticancer drugs (paclitaxel) [49,50]. CDs can be previously modified to be combined with other monomers. A good example of this is the acrylated-CDs. After modification they can be incorporated into acrylic acid polymers [43]. This type of polymer has been used to delivery doxorubicin (DOX) and paclitaxel (PTX) [51,52]. However, all these crosslinkers could present toxicity problems that can limit the biomedical applications and additionally present environmental problems. In order to overcome this limitation, citric acid has been proposed as a “green” crosslinker [43]. This molecule was used to crosslink γ-CD to obtain hydrogels capable of delivering anthracycline.

## 3. Delivery Applications

The hydrogel types described in the previous sections have potential to be used as drug delivery systems. Taking into account the number of hydrophobic drugs in the market and currently in development [11,53], the development of hydrogels for hydrophobic drug delivery could present multiple advantages for patients and clinicians. The main delivery routes where these hydrogels have been used are oral, subcutaneous and transdermal delivery [54,55,56]. Table 1 summarises the main applications of hydrogels that will be discussed in the following sections.

### 3.1. Sub-Cutaneous Hydrogel Drug Delivery

Thermosensitive hydrogels have been widely investigated, because they can achieve localized drug delivery without the need for invasive surgical insertion or removal of the drug delivery system. Most are injectable systems, which undergo transition from free flowing sol at room temperature, to a polymerised gel at physiological temperature [59,84], allowing sustained drug release in situ. The gel phase will only appear if the polymer concentration is above the critical gel concentration (CGC) [85]. CGC is often inversely related to the polymer’s molecular weight [85]. This type of system offers advantages such as: reduced systemic toxicity as a result of localized delivery; improved patient compliance and comfort [86]; and easy administration via injection [58]. However, challenges with this type of system include: difficulty in achieving high drug loading with poorly soluble hydrophobic drugs; problems with stability; and problems achieving long term sustained drug release [59]. Several strategies have been employed to overcome these issues. The first one was the synthesis of novel temperature sensitive biomaterials or optimization of classic polymers such as PCL-PEG-PCL and Pluronic F127 [59,87]. Cohn et al. investigated the effect of chain extension on the mechanical and rheological properties of the thermosensitive polymers and found that polymers with enhanced mechanical properties and long term stability could be produced [87].

The next strategy was the design of a particle–hydrogel combination system to entrap liposomes, nanoparticles or microspheres within the thermosensitive gel. Nie et al. proposed a system comprising a PTX containing liposomes dispersed within a thermosensitive hydrogel and concluded that the addition of a drug containing liposomes can offer improved stability and sustainability for the delivery of hydrophobic drugs [88]. The last approach was the construction of a mixed micelle gel consisting of a thermosensitive polymer and a surfactant. Yang et al. investigated the addition of the surfactant Tween 80 and found that its addition could improve the physical stability, drug release and solubility of highly hydrophobic drugs from thermosensitive polymer systems [89]. One concern with this type of system is the possibility of large amounts of the drug being released before the sol-gel transition has occurred [86]. To minimize this, ideally the lag time between injection of the sol and formation of the gel should be as small as possible. This can be controlled by changing the molecular weight of the polymer or by the addition of a surfactant [86].

Most thermosensitive hydrogel forming polymers are comprised of block polymers containing poly(ethylene glycol) (PEG) coupled to hydrophobic polymer blocks [10]. The blocks are composed of A and B blocks arranged as ABA or BAB [10]. PEG A blocks ensure that the matrix is biocompatible and increases water solubility, whereas, the B block, e.g., poly(propylene oxide) or poly(lactic acid) (PLA), gives the matrix hydrophobicity [10].

Regel^®^ is an example of a thermosensitive hydrogel, consisting of a copolymer comprised of PLGA and PEG arranged into PLGA-PEG-PGLA triblocks [10,86,90]. Regel^®^ has the ability to increase the solubility of hydrophobic compounds because it possesses a hydrophobic core and it is therefore capable of delivering small hydrophobic molecules [90]. It has been shown to be capable of successfully delivering ketoprofen, a hydrophilic drug, and spironolactone, a relatively hydrophobic drug for two weeks and two months, respectively, into a 37 °C aqueous environment [10,23]. Gao et al. investigated the intratumoral delivery of docetaxel (DTX) from a PLGA-PEG-PLGA triblock copolymer and found that the solubility of DTX was increased more than 3000-fold and sustained release of the drug for more than three weeks was achieved [57]. This system was found to be more efficient than intravenous injections of DTX, with one intratumoral injection being equivalent to three intravenous injections. Moreover, it delivered the treatment with lower toxicity [57]. Commercial products utilising the Regel^®^ system have been formulated to deliver paclitaxel (Oncogel^®^), human growth hormone and interleukin-2 (Cytoryn) [86]. Pluronic^®^ F127 is another widely studied, FDA approved, thermosensitive hydrogel that is used for this application [59,88]. It consists of poly(ethylene oxide) (PEO) and poly(propylene oxide) (PPO) in a PEO-PPO-PEO triblock [10,86,91].

**Figure 5:**
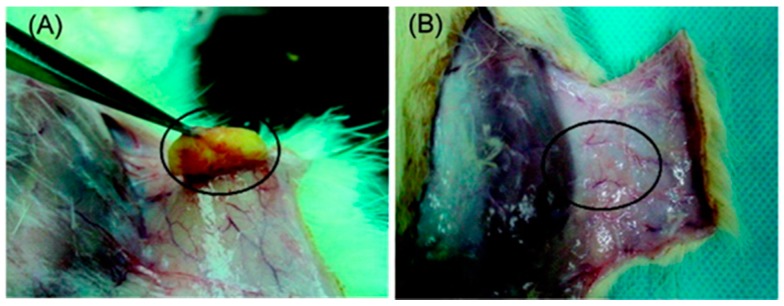
PCL-based copolymer gel at 20 wt % (circle to mark the gel) formed in a rat, four weeks after injection (**A**), and Pluronic 20 wt % gel after two days (**B**). Reproduced with permission from [61]. Copyright (2007) American Chemical Society.

PCL is another relevant biodegradable polymer in this field of research. PEG-PCL di-blocks or tri-blocks can be synthesised. Studies comparing PEG-PCL copolymers to Pluronic F127 have found that PEG-PCL is capable of retaining its structural integrity for longer than the Pluronic F127 [61,92] (Figure 5). The ability of the two systems to deliver fluorescein isothiocyanate labelled bovine serum albumin (BSA FITC) was compared by Hyun et al., and it was observed that a more sustained release occurred from the PEG-PCL copolymer than from the Pluronic F127 [61]. This slow degradation of PCL based hydrogels is as a result of PCLs high crystallinity and hydrophobicity [93]. However, this extremely slow degradation may not be desirable. Crystallinity, and therefore, degradation time may be reduced by the incorporation of lactic acid or glycolic acid, or by addition of a cyclic ether group such as 1,4,8-trioxa[4.6]spiro-9-undecanone [93]. 

In order to increase the degradation rates, PCL could be substituted with a polymer with lower crystallinity. Poly(valerolactone) (PVL), for example, undergoes a faster rate of degradation than PCL as a result of aqueous hydrolysis and lipase degradation and generates by-products which can be more readily eliminated than those of PCL [94]. Polypeptide based thermosensitive systems have been investigated and found to have a much lower CGC than traditional polyester based ones [62]. Cheng et al. investigated the delivery of PTX in a thermosensitive hydrogel made from poly(γ-ethyl-l-glutamate)-poly(ethylene glycol)-poly(γ-ethyl-l-glutamate) (PELG-PEG-PELG) triblock copolymers and found that the hydrogel lasted in the subcutaneous tissue for 21 days and was able to successfully deliver PTX and suppress tumour growth in a rat model [62].

### 3.2. Oral Delivery

Oral drug delivery is the preferred route of drug administration due to its simplicity. It presents advantages over intravenous administration such as: lower price [95] and better patient compliance [96]. It has some drawbacks as the harsh conditions of the gastrointestinal (GI) tract can degrade drugs. Moreover, drugs administered using this route suffer the first-pass effect.

Hydrogels could enhance oral administration providing controlled release over prolonged periods of time while protecting the drug from the harsh conditions in the stomach [97]. Hydrogels can be designed containing pH-responsible groups that are capable of releasing their cargoes in specific parts of the gastrointestinal (GI) tract [98]. The ideal example are hydrogels containing PAA chains. Hydrogels containing PAA can be ionized where the pH is superior to the pKa of the PAA acid groups (around 5) yielding swellable hydrogels [98]. On the other hand, at a pH lower than the pKa of the PAA, the hydrogel forms a compact collapsed structure [98]. Moreover, it has been reported that PAA chains can inhibit the proteolytic enzymes in the GI tract [99] providing extra protection for drugs loaded inside the hydrogel.

Bromber et al. synthesized a PAA-based hydrogel by combining an ampiphilic PEG- poly(propylene glycol) block copolymer (Pluronic^®^) with PAA to develop a stimuli responsive amphiphilic hydrogel [68]. The presence of Pluronic provided temperature responsive properties to the hydrogel while the PAA provided pH responsive properties. This system was tested as an oral delivery system for hydrophobic compounds such as camptothecins, steroid hormones, DOX, mitomycin C and mitoxantrone. A modified version of these hydrogels containin methacrylates and methyl methacrylate (MMA) was developed by Caldorera-Moore et al. [69]. This system was able to deliver a model hydrophobic compound (fluorescein) using an artificial intestinal lining. The presence of hydrophobic MMA domains increased the drug loading while reducing the pH responsiveness of the material [69]. Using a different strategy, Schoener et al. developed amphiphilic interpenetrating networks made of PMA grafted with PEG tethers and hydrophobic poly(butyl acrylate) chains for the local delivery of fluorescein in colon [70].

PMMA nanoparticles containing hydrophobic compounds showed good release capabilities using a model hydrophobic compound (fluorescein) [37]. Consequently, the system was optimized for the release of DOX to treat colon cancer [72]. The presence of nanoparticles within the structure allowed more sustained release processes at higher pHs as they shielded ionic repulsion between ionized carboxyl groups and reduce the mobility of the polymer chains [37]. As an alternative for PAA-based carriers poly(2-hexenoic acid), a PAA based derivative, was crosslinked using a biodegradable polypeptide for oral delivery of insulin. This system is biodegradable as opposed to PAA hydrogels [100]. This hydrogel showed a reduced swelling/release ratio under stomach conditions. On the other hand, when placed under intestinal conditions the swelling and the release rate increased significantly. The systems were tested in an in vivo diabetic rat model. The administration of the insulin-loaded hydrogel to the animals showed a hypoglycemic effect within seven hours, with glucose level reduced close to 70% continuously [100]. Another biodegradable alternative to PAA are hydrogels made of an anionic pseudo-peptidic polymer, poly(l-lysine isophthalamide). This compound is amphiphilic and pH responsive. The use of this compound to prepare hydrogels for oral delivery of hydrophobic drugs was tested by Watkins et al. [101].

**Figure 6:**
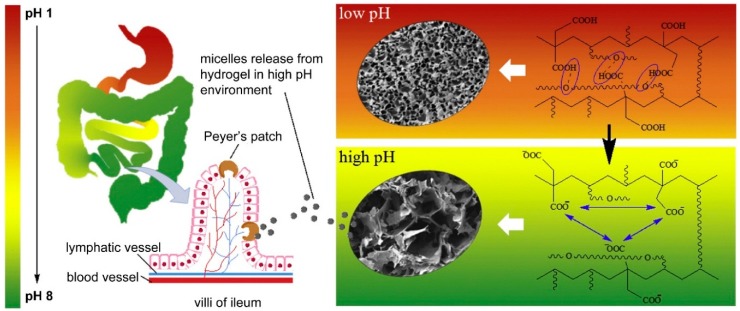
Scheme of micelles delivery in gastrointestinal tract. The pH value increases from 1 (in stomach) to 8 (in ileum). The hydrogels shrink in low pH environment, while in a high pH environment, they swell and release micelles. The micelles are absorbed in ileum through Peyer’s patches. Reproduced with permission from [83].

Hydrogels containing hydrophobic domains can be adapted to achieve delivery of hydrophobic drugs through the epithelial layer of the GI tract into blood stream. Wang et al. developed a micellar hydrogel capable of delivering docetaxel after oral administration for breast cancer treatment. The system contained PCL-PEG-PCL micelles loaded with docetaxel included in a poly(ethylene glycol) methyl ether methacrylate and itaconic acid (pH sensitive fragment) hydrogel [71] (Figure 6). The system improved the oral bioavailability of docetaxel about 10 times (around 75%) when compared with docetaxel micelles alone. Moreover, the system was able to inhibit tumour growth in a breast cancer model reducing systemic toxicity compared with the conventional intravenous treatment when applied to a mouse animal model [71]. 

### 3.3. Ocular Delivery

Hydrogels containing hydrophobic drugs have been used for the delivery of hydrophobic drugs. However, ocular delivery of hydrophobic drugs using hydrogels has not been extensively explored. The two main possible scenarios for ocular delivery are implantable systems or medicated contact lenses. For example, Lu et al. described a PEG/silica hydrogel system capable of delivering hydrophilic and hydrophobic drugs [76]. This system was designed to be injected into the eye to create a depot for the sustained delivery of dexamethasone. In this work, multiarm poly(ethylene glycol) (PEG)/silica hydrogels were synthesized. The synthetic process was carried out by hydrolysis and condensation of poly(4-arm PEG silicate) using the sol-gel method [76]. Similarly, Xi et al. developed an injectable thermosensitive hydrogel based on poly(trimethylene carbonate) and Pluronic F127 to deliver mitomycin C [77]. The hydrogels provided full drug release within 25 days.

**Figure 7:**
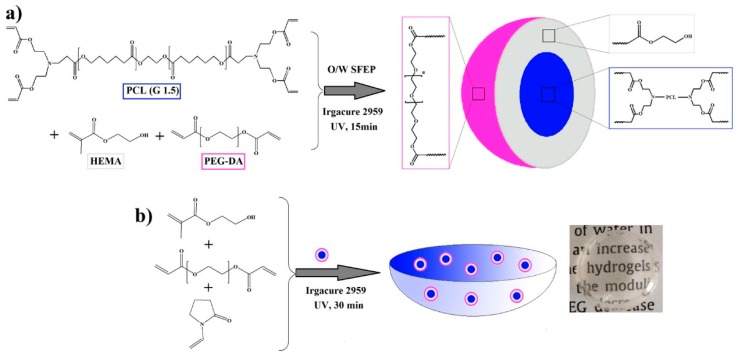
Schematic illustration of surfactant-free emulsion polymerization for nanoparticles synthesis (**a**) and nanoparticle-loaded hydrogel Preparation (**b**). Reproduced with permission from [36]. Copyright (2016) American Chemical Society.

Several examples of hydrogel-based contact lenses can be found in the literature for the delivery of hydrophobic drugs. Silicone-based hydrogels have been used to develop medicated contact lenses for hydrophobic drug delivery [73]. Atropine was selected as a model hydrophobic drug. The silicone hydrogel contact lenses were able to provide up to two weeks of sustained release of this molecule. In a similar way, Gulsen et al. used microemulsions loaded within HEMA hydrogels for ocular drug delivery [55]. In this case, HEMA hydrogels were loaded with a lidocaine emulsion and the system provided up to eight days of sustained release. The same strategy was followed by Kapoor et al. showing that HEMA hydrogels were capable of delivering when loaded as a microemulsion Cycloxporine A [74]. However, the system failed to deliver other hydrophobic compounds such as dexamethasone and dexamethasone 21 acetate [74]. In a similar way, Nasr et al. developed hydrogel-based contact lenses containing nanoparticles for the prolonged delivery of loteprednol etabonate [36] (Figure 7).

Finally, hydrogels containing CD have been used for ocular hydrophobic drug delivery. Rosa dos Santos et al. showed that hydrogel-based contact lenses containing CDs were able to deliver diclofenac for 25 days [75]. For this purpose, poly(hydroxyethylmethacrylate) hydrogels were synthesized by copolymerization with glycidyl methacrylate. Subsequently, β-CD was grafted to the network by reaction with the glycidyl groups [75].

### 3.4. Transdermal Delivery

The transdermal route has been proven to be a good alternative to oral drug delivery. It is painless and can be self-administered providing a high patient compliance. Additionally, drugs administered using this route avoid the first-pass effect and the harsh conditions of the GI tract [102]. However, the main drawback of the transdermal route is that only a few drugs fulfill all the physicochemical requirements to be able to permeate passively through the skin. The use of pH-sensible hydrogels capable of loading hydrophobic drugs have the potential to treat different skin conditions. A pH imbalance is one of the main causes leading to skin inflammation and acne. In normal conditions the skin surface has a pH ranging from 5 to 6. Changes in the skin pH can potentially lead to compromise the barrier function of the *stratum corneum* [103]. Kwon et al. described a new type of hydrogels made of hyaluronic acid and cellulose, as the hydrophobic molecule, to deliver an antimicrobial therapeutic agent for acne growth inhibition (isoliquiritigenin) [78]. This type of hydrogel presented pH responsive properties and it was able to maximize the cargo release at around pH 7. The colony formation of acne presents the peak of activity at this pH. The drug was able to permeate the skin barrier via the follicular pathway due to the hydrogel-assisted swelling of the skin [78].

Hydrogels have been used as a method to increase the viscosity of the low-viscosity formulations to keep contact with the skin surface for longer periods of time. Micro and nanoparticle systems containing poor water soluble compounds were incorporated in hydrogel materials to be delivered locally and systemically. A microemulsion based hydrogel system (MBH) has been prepared for the topical delivery of penciclover anti-viral drug. The MBH system was prepared by mixing microemulsion with Carbomer 940 gelling agent which swelled by water content of the emulsion to give viscous hydrogel suitable for topical application. When compared to the commercially used cream, the system showed comparable ability to increase the permeation of penciclovir to the dermis and epidermis [79].

Natural compounds have been incorporated and studied for their wound healing activities. Curcumin has been reported to be active in wound healing orally and topically. The efficacy of curcumin is limited by its poor water solubility. Gong et al. described a curcumin-loaded in situ gel-forming sustained drug delivery system to promote wound healing. In this delivery system, curcumin was dispersed in poly(ethylene glycol)-poly(caprolactone) (PEG-PCL) copolymer as an amorphous molecule [80]. Upon evaporation, the dispersion self-assembled into micelles with curcumin encapsulated inside. The prepared micelles were then incorporated in a thermosensitive hydrogel system composed of PEG-PCL-PEG copolymer. This prepared system showed a temperature affected sol-gel activity, thus, it could adhere to the wounds at skin temperature. This curcumin-loaded in situ forming hydrogel showed a sustained release of 40.1 ± 2.5% of its load of curcumin over 14 days. Chen et al. incorporated solid lipid nanoparticles (SLNs) containing natural astrogaloside IV for wound healing and anti-scar activity of this compound into carbomer 934 gel [81]. After application to the wounds in vivo, the SLN particles entered the cells through endocytosis mechanism and resulted in acceleration of wound re-epithelialization when compared to blank control. 

**Figure 8:**
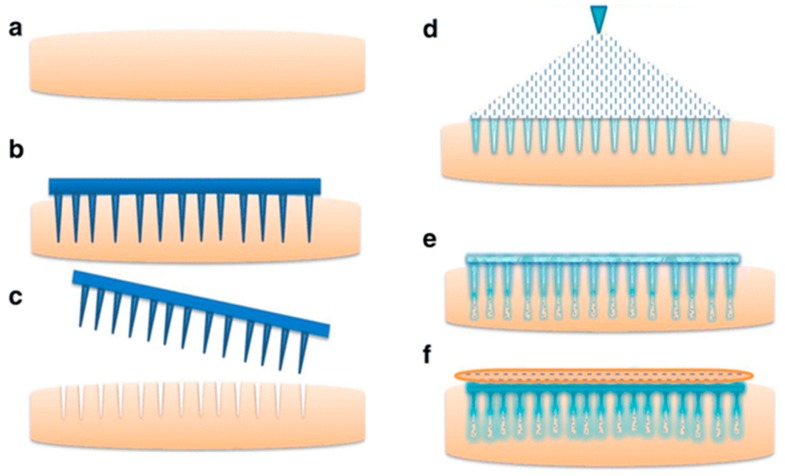
Schematic representation of formation and delivery of drug from in situ forming hydrogel microneedles (**a**). Non-porated skin (**b**), skin porated with maltose microneedles (**c**), pore formation after removal of maltose microneedles (**d**), application of drug-poloxamer solution (**e**). Flow of drug-poloxamer solution into the porated site of skin (**f**) transition of drug-poloxamer solution to gel state at skin temperature (32 °C) to form in situ hydrogel microneedles. Reproduced with permission from [95].

Hydrogels have been used to deliver poorly water-soluble compounds transdermally to elicit a systematic effect as well. Bhashkar et al. developed SLN and nanostructured lipid carrier (NLC) enriched hydrogel systems to provide a controlled delivery of the antihypertensive nitrendipine transdermally [82]. Hydrogels were prepared from four formulations; Carbopol 934, xanthan gum, hydroxy propyl cellulose (HPC) or chitosan. Hydrogels were combined with the SLNs and NLS particles through high-speed stirring. Plasma concentrations of nitrendipine after in vivo studies in rats proved the sustained and continued drug release of for 24 h when compared to oral administration. Moreover, a novel technique has been utilised to deliver Methotrexate drug molecule transdermally. In situ forming hydrogel microneedles were prepared using thermosensitive copolymer (poloxamer P407) that transition from a solution at room temperature to a gel at skin temperature (32 °C). More specifically, this technique used maltose microneedles to microporate human dermatomed skin, followed by application of poloxamer-based drug formulation where the solution flowed inside the created microchannels, transitioned into gel at skin temperature, and attained the shape of microneedle as represented in Figure 8. In vitro results showed that these in situ formed hydrogel microneedles delivered the encapsulated drug in a sustained fashion [83].

## 4. Conclusions

Significant developments have been made to improve the properties of hydrogels for drug delivery. The literature covers the use of this type of materials for a wide variety of purposes. Hydrogels have been extensively studied for the delivery of hydrophilic compounds. However, scientists should address a series of challenges related with hydrogel materials. One of the main challenges is to improve their compatibility with hydrophobic compounds.

The present article summarizes the latest progress made to develop hydrogels capable of delivering poorly water-soluble drugs. This is an important area of research due mainly to the interesting properties of hydrogel materials and to the increasing presence of hydrophobic drugs in the market. This type of materials has shown potential to deliver hydrophobic compounds, however there are not many clinical trials describing the use of these materials. One of the possible reasons is that the development of these systems is not simple as complex synthetic steps are required. Consequently, to scale up the manufacturing process of such hydrogels is challenging. Additionally, some of the reagents are potentially toxic limiting the biomedical applicability of the materials. Further work should be carried out to develop hydrogel systems capable of delivering hydrophobic compounds in a cheap, safe and scalable way.

There are still several challenges to be addressed before seeing more hydrophobic drug delivery systems based on hydrogels applied clinically. The safety of the material is crucial and as mentioned previously, the synthetic procedure should be designed accordingly. Reagents that could be potentially toxic should be replaced by molecules with a well-established safety profile. Moreover, these type of hydrogel systems should be prepared using FDA-approved polymers/reagents. This will significantly speed up the regulatory clearance of the new materials allowing a quicker pathway to the market. Finally, the last aspect that should be considered is the manufacturing process, which ideally should be cheap and scalable. Some of the hydrogel systems described in this article present synthetic steps that will be difficult to scale up. A good example are the hydrogels that contain nanoparticles within their structure. These nanoparticles should be prepared prior to the hydrogel synthesis making the overall process more complex. The complexity of the synthetic step will be translated in a higher manufacturing cost.

## Figures and Tables

**Table 1:** Main examples of delivery of therapeutics using hydrogels containing hydrophobic moieties.

Hydrogel System	Agent Delivered	Target Treatment	Route Administration	References
PLGA-PEG-PLGA	Docetaxel	Lung cancer	Subcutaneous	[57,58]
PLGA-PEG-PLGA	Paclitaxel	Breast cancer	Subcutaneous	[59]
Modified PCL-PEG-PCL	Doxorubicin	Liver cancer	Subcutaneous	[60]
PEG-PCL diblock	Isothiocyanate labelled bovine serum albumin	-	Subcutaneous	[61]
Poly(γ-ethyl-l-glutamate)-PEG-poly(γ-ethyl-l-glutamate)	Paclitaxel	Liver cancer	Subcutaneous	[62]
Peptide-graphene hybrid	Doxorubicin	Liver cancer	Subcutaneous	[63]
Poly(l-lysine)-PAladiblock peptide	Tamoxifen	Central nerve system	Subcutaneous	[64]
Nucleobase-terminated PEG with CD	Doxorubicin	Cervical cancer	Subcutaneous	[65]
Vitamin E-PEG-Vitamin E	Herceptin	Breast cancer	Subcutaneous	[66]
Vitamin D-PEG-Vitamin D	Avastin	Colorectal cancer	Subcutaneous	[67]
PAA-Pluronic Hydrogel	Camptothecins, steroid hormones, doxorubicin, mitomycin C and mitoxantrone	-	Oral	[68]
PMMA/PAA-based hydrogel	Fluorescein	-	Oral	[69]
PMA/PEG/poly(butyl acrylate)-based hydrogel	Fluorescein	-	Oral	[70]
PCL-PEG-PCL	Docetaxel	Breast cancer	Oral	[71]
Hydrogels containing PMMA nanoparticles	Doxorubicin	Colon cancer	Oral	[72]
Silicone-based hydrogel	Atropine	-	Ocular (contact lenses)	[73]
HEMA-based hydrogels containing microemulsions	Lidocaine	-	Ocular (contact lenses)	[55]
HEMA-based hydrogels containing microemulsions	Cycloxporine A	-	Ocular (contact lenses)	[74]
HEMA-based hydrogels containin nanoparticles	Loteprednol etabonate	-	Ocular (contact lenses)	[36]
HEMA-based hydrogels containing CDs	Diclofenac	Ocular inflammatory disorders	Ocular (contact lenses)	[75]
PEG/silica hydrogel	Dexamethasone	-	Ocular (injectable implant)	[76]
Poly(trimethylene carbonate)/Pluronic F127 hydrogel	Mitomycin C	-	Ocular (injectable implant)	[77]
Cellulose/hyaluronic acid-based hydrogel	Isoliquiritigenin	Anti-microbial therapy	Topical	[78]
Carbomer 940-ased gel containin a microemulsion	Penciclovir	Anti-viral therapy	Topical	[79]
PEG-PCL-based hydrogel	Curcumin	Wound healing	Topical	[80]
Carbomer 940-based gel containing solid lipid nanoparticles	Astrogaloside IV	Wound healing	Topical	[81]
Carbopol 934, xanthan gum, hydroxy propyl cellulose (HPC) and chitosan based hydrogels containing nanostructured lipid carriers	Nitrendipine	Hypertension	Transdermal	[82]
Pluronic-based hydrogel	Methotrexate	Solid tumours	Transdermal	[83]

Note: If target treatment was not disclosed is because the study was designed as a proof of concept.
